# Positive symptom phenotypes appear progressively in “EDiPS”, a new animal model of the schizophrenia prodrome

**DOI:** 10.1038/s41598-021-83681-4

**Published:** 2021-02-22

**Authors:** Alice Petty, Xiaoying Cui, Asad Ali, Zilong Du, Sunil Srivastav, James P. Kesby, Deniz Kirik, Oliver Howes, Darryl Eyles

**Affiliations:** 1 Queensland Brain Institute, University of Queensland, Brisbane, QLD 4072 Australia; 2 MRS London Institute of Medical Sciences, Hammersmith Hospital, London, UK; 3 BRAINS Unit, Department of Experimental Medical Science, Lund University, 22184 Lund, Sweden; 4 Department of Psychosis Studies, Institute of Psychiatry, Psychology & Neuroscience, King’s College London, London, UK; 5 Institute of Clinical Sciences, Faculty of Medicine, Imperial College London, London, UK; 6 Queensland Centre for Mental Health Research, Wacol, QLD 4076 Australia

**Keywords:** Cellular neuroscience, Molecular neuroscience, Behavioural methods, Psychiatric disorders, Experimental models of disease, Preclinical research, Translational research

## Abstract

An increase in dopamine (DA) synthesis capacity in the dorsal striatum (DS) during the prodromal stage of schizophrenia becomes more pronounced as patients progress to the full disorder. Understanding this progression is critical to intervening in disease course. We developed an animal model—Enhanced Dopamine in Prodromal Schizophrenia (EDiPS)—which uses a genetic construct to increase DA synthesis capacity in the DS of male rats. We assessed pre-pulse inhibition (PPI) and amphetamine (AMPH)-induced locomotion (0.6 mg/kg) in EDiPS animals longitudinally after post-natal day 35 (when the EDiPS construct is administered). We also assessed their response to repeated acute restraint stress. In adult EDiPS animals, we measured baseline and evoked extracellular DA levels, and their stereotyped responses to 5 mg/kg AMPH. AMPH-induced hyperlocomotion was apparent in EDiPS animals 6-weeks after construct administration. There was an overall PPI deficit in EDiPS animals across all timepoints, however the stress response of EDiPS animals was unaltered. Adult EDiPS animals show normal baseline and potassium-evoked DA release in the DS. These findings suggest that key behavioural phenotypes in EDiPS animals show a progressive onset, similar to that demonstrated by patients as they transition to schizophrenia. The EDiPS model could therefore be used to investigate the molecular mechanisms underlying the prodrome of schizophrenia.

## Introduction

Prior to the diagnosis of schizophrenia, patients typically display attenuated positive, cognitive, and negative symptoms, during a period known as the prodrome^1^. These symptoms increase in severity until they meet the clinical criteria for schizophrenia. It is possible to identify those people who are at high risk of developing schizophrenia (an At-Risk Mental State; ARMS), and who may therefore be in the prodromal stage of the disorder^2,3^. Only approximately 20% of ARMS subjects will ultimately transition to schizophrenia^4^. ARMS patients are often identified in late adolescence/early adulthood^5^, and currently 20 registered intervention trials in ARMS patients have been completed. Unfortunately, recent meta-analyses show that no intervention was effective at improving symptoms in ARMS patients^6–8^, or at reducing rates of transition at either a 6- or 12-month follow-up point^9^. An improved understanding of the molecular basis of the prodrome is critical to inform future interventions designed to diminish symptom severity, or to prevent disease onset.

One robust finding in patients with schizophrenia is an increase in dopamine (DA) synthesis capacity in the dorsal striatum (DS)^10^. This abnormality is also evident in prodromal patients^11–14^. Importantly, DA uptake increases further with disease progression^15^. The increase in pre-synaptic DA synthesis capacity as patients transition to clinical schizophrenia suggests that this abnormality may be a core feature of the course of disease. It further suggests that late-developing DA systems in the DS may be a promising target for intervention.

Animal models are required to understand the neurobiological course of schizophrenia and to test novel treatment approaches. This is especially the case in ARMS cohorts, where trialling novel interventions may be unnecessarily risky for the 80% of patients who would not have transitioned to schizophrenia. Enhanced Dopamine in Prodromal Schizophrenia (EDiPS) is an animal model designed specifically to understand the neurobiology of increased DA synthesis capacity in the prodrome^16^. In this model, an adeno-associated virus (AAV)-packaged genetic construct is delivered at post-natal day 35 (P35). P35 is considered a pre- or peri-pubertal period in a rat^17,18^. There is evidence that, like humans, rodent brains undergo substantial structural and neurochemical modulation during the transition to adulthood^17,18^. The AAV construct codes for the DA-synthesizing enzymes tyrosine hydroxylase (TH) and guanosine triphosphate (GTP) cyclohydrolase 1 (GCH1). The construct is delivered into the substantia nigra pars compacta (SNpc), a dopaminergic region in the midbrain which projects preferentially to the DS^19^. Adult EDiPS animals show an enhanced release of DA preferentially in the DS in response to 0.6 mg/kg amphetamine (AMPH)^16^. This mimics the increased DA synthesis capacity seen so robustly in patients^10^.

EDiPS is a useful model to investigate the effects of increased DA synthesis and release within the DS on brain connectivity and function, however this model was also designed to study the prodromal phase of schizophrenia. Adult EDiPS animals display increased AMPH-induced hyperlocomotion and deficits in pre-pulse inhibition (PPI)^16^. Therefore, a primary objective of this study was to establish whether these behaviours gradually emerge across adolescence, thus recapitulating the time-course over which the symptoms of schizophrenia emerge in patients.

Secondary objectives in this study were threefold. (A) Stress-sensitivity can predict the severity of prodromal symptoms, as well as the likelihood of transition to the clinical disorder^2,20,21^. Therefore, an additional aim of this study was to assess whether the response to stress increases in parallel with the emergence of positive symptom phenotypes in EDiPS animals. (B) There is evidence from three PET studies to date which indirectly suggests that synaptic DA is elevated at baseline in patients with schizophrenia^10^. Another secondary aim of this study was to investigate whether baseline extracellular DA and/or evoked DA release is elevated in adult EDiPS animals. (C) High dose AMPH induces stereotyped repetitive behaviours^22^ and there is robust evidence that such behaviours are modulated primarily by DA in the DS^23^. Therefore, a final secondary aim of this study was to examine whether such behaviours are exacerbated in adult EDiPS animals.

## Methods

### Animals and housing

**Figure 1:**
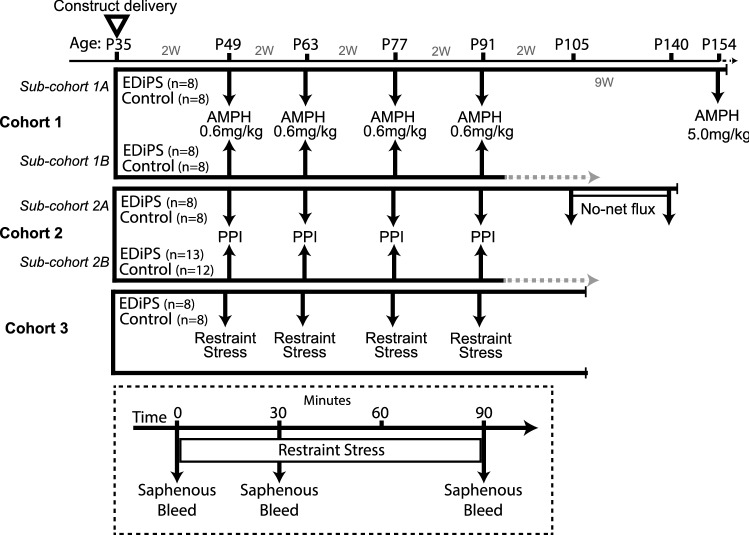
Timeline of experimental testing. All animals were injected with the EDiPS or control construct at age P35. Longitudinal testing began 2 weeks later and occurred every two weeks for 4 timepoints. Of the cohort examined for AMPH-mediated locomotion, a sub-cohort went on to be tested with 5 mg/kg AMPH. Of the cohort examined for PPI, one sub-cohort went on to undergo no-net flux microdialysis. A separate cohort was used to assess the response to restraint stress. The inset shows the protocol used to assess the response to restraint stress; saphenous bleeds were taken before (t = 0), during (t = 30) and immediately after (t = 90) the restraint stress procedure. Plasma from this blood was assayed for levels of corticosterone. *AMPH* Amphetamine, *PPI* pre-pulse inhibition.

Male Sprague–Dawley rats were acquired at age P21 from the Animal Resources Centre (ARC, South Australia), and pair-housed with ad libitum food and water. Of the animals which underwent repeat 0.6 mg/kg AMPH, one sub-cohort were used for behavioural assessment after 5 mg/kg AMPH (Fig. 1). Of those animals which underwent PPI testing, one sub-cohort were used for microdialysis. A separate cohort of animals was used to assess the effect of repeated restraint stress. Progressive tests were performed every 2 weeks for 4 timepoints, starting 2 weeks following construct delivery. All animal procedures were approved by The University of Queensland Animal Ethics Committee, under the guidelines of the National Health and Medical Research Council of Australia. This study was carried out in compliance with the ARRIVE guidelines for animal research.

### Generating EDiPS animals

Detailed methods regarding the generation of EDiPS animals have been published previously^16^. The EDiPS construct is an AAV2/1-packaged construct containing the human tyrosine hydroxylase (huTH) and human GTP cyclohydrolase 1 (huGCH1) genes, each driven by a human synapsin-1 promoter^16^. This construct is untagged. The construct was delivered bilaterally to the substantia nigra pars compacta of a P35 animal through a stereotaxic surgery protocol. Briefly, animals were anaesthetised, placed in the stereotaxic frame, and a sagittal incision made to reveal the skull. Bilateral holes were drilled for injection of the construct at (from Bregma) Anterior–Posterior: − 5.2 mm, Lateral–Medial: − 2.2 mm, Dorsal–Ventral (from dura): − 7.6 mm. The construct was delivered slowly using a pulled glass micropipette attached to a manual syringe. Following the injection, the incision site was sutured, and animals were allowed to recover individually housed for 48 h, before returning to pair-housing. Behavioural testing was initiated 2 weeks following construct delivery. An additional animal was injected with the EDiPS construct, and euthanised 2 weeks later, to assess construct expression in the midbrain and striatum at this early time-point (see Supplementary Fig. S1). These sections were compared to those from an animal taken 8-weeks following construct delivery. At the 2-week timepoint, the construct is highly expressed in the midbrain, but not the striatum. However, 8-weeks following construct administration, expression is evident in both the midbrain and striatum.

### Longitudinal 0.6 mg/kg AMPH-induced hyperlocomotion

AMPH-induced locomotion was assessed using matt black 60 × 60 × 60 cm chambers, as described previously^16^. Baseline locomotion was recorded for 30 min, then animals were removed and injected with 0.6 mg/kg dexamphetamine (AMPH) intraperitoneally. Animals were then placed back into the chamber and recorded for a further 90 min. Distance travelled was calculated using EthoVision software (Noldus, Ver. 13.0).

### Longitudinal pre-pulse inhibition (PPI)

The PPI protocol used was identical to that described previously^16^. Briefly, pre-pulses at three different intensities (74, 78, 86 dB) were played at a variety of intervals (8, 16, 32, 64, 128, 256 ms) prior to the startle pulse (120 dB) to assess the suppression of the startle response. The background noise level was 70 dB. The median of each trial type was used for analysis. Only PPI results using the 86 dB pre-pulse were analysed, since this resulted in the greatest degree of inhibition in control animals. This data-set reflects the average %PPI for all intervals for which the 86 dB pre-pulse stimulus was trialled. The acoustic startle response (ASR) was adjusted for weight gain during adolescence by dividing the ASR to each startle amplitude by the weight of each animal at each timepoint.

### Longitudinal restraint stress

Restraint induces a robust activation of the hypothalamus–pituitary–adrenal (HPA) axis in rodents^24,25^. Animals were restrained for 90 min using a plastic mesh tube which was placed under bright light (~ 1000 lx). Blood was collected from the saphenous vein for assessment of corticosterone (CORT) levels at 3 times during the 90-min test (see Fig. 1). Blood was collected into ethylenediaminetetraacetic acid (EDTA)-coated Microvette tubes (Sarstedt Inc.). This test occurred on a single day at 2-, 4-, 6- and 8-weeks following construct delivery.

### Quantification of corticosterone (CORT)

CORT levels were quantified by an in-house LC–MS/MS protocol used previously in the group^26^. Plasma was dispensed in 1:1 acetone:ethanol, and 200μL of deuterated corticosterone –[^2^H_4_] was added as an internal standard. Samples were transferred to conditioned SPE cartridges (Strata C18-E (55uM, 70A) 50 mg/1 mL) and washed with 35% methanol. The analytes were eluted, dried and reconstituted with 50% methanol. This extract was injected onto a UPLC column [Phenomenex Kinetex 1.7u XB-C18 100A (50 × 2.1 mm)] and eluted using a gradient method with mobile phase A (mpA) = 0.1% aqueous formic acid and mobile phase B (mpB) = 0.1% formic acid in 95:5 acetonitrile: water. The detection on an API5500-QTrap (Applied Biosystems) used positive ion MRM mode with electrospray ionization. The mass-spectrometer potentials were as follows: for corticosterone *m/z* = 347.1 → 329.1, declustering potential, (DP) = 120 V, collision energy (CE) = 23 V; for corticosterone –[^2^H_4_] 351 → 333 DP = 120 CE = 18. Differential quality control samples were prepared by spiking rat serum with 75 nM of corticosterone. Absolute CORT values were analysed to examine whether baseline levels of CORT were altered between EDiPS and control animals, or whether they were affected by repeat testing. Stress responsivity was calculated as area under the curve across the 0-, 30- and 90-min timepoints.

### Baseline extracellular DA and KCl-induced DA release

Microdialysis was performed under isoflurane anaesthesia. A no-net flux technique was used to establish baseline extracellular DA levels. A normal microdialysis protocol was then used to examine potassium (KCl)-induced DA release. A 3 mm probe (CMA 12 Elite, Harvard Apparatus) was inserted into the right hemisphere DS at: A-P (from bregma): + 0.6 mm, M-L: − 2.6 mm, D-V (from dura): − 5.5 mm. Once inserted, the probe was perfused with regular artificial Cerebral Spinal Fluid (aCSF) containing 154.1 mM Cl^−^, 147 mM Na^+^, 2.7 mM K^+^, 1 mM Mg^2+^, and 1.2 mM Ca^2+^ (pH approximately 7.4) at 1 μl/min. Each sample bin was 15 min. The last 2 of the first 4 baseline samples was used for analysis (the 0 nM value for no-net flux calculations). Following baseline, four separate aCSF solutions with DA concentrations of 2 nM, 5 nM, 10 nM and 20 nM were infused in a random order. Three bins were collected for each concentration, with the first bin excluded from analysis. Following the last concentration of DA, the perfusate was changed back to regular aCSF for one hour. Then, a 100 mM potassium (K^+^; KCl) solution was perfused for 15 min (1 bin), before being changed back to regular aCSF for a further 3 bins. Dialysate was collected into tubes containing 3.75 μl of 0.1 M perchloric acid and were immediately assayed for DA and other analytes.

### Quantification of DA, serotonin and metabolites

Dialysate was injected into an HPLC system (Agilent Technologies, Inc., CA, USA). Two different mobile phases were used. One (containing 50 mM Citric Acid.H_2_O, 25 mM NaH_2_PO_4,_ 1 mM EDTA, 1.6 mM octane sulfonic acid in 10% acetonitrile; pH adjusted to 5.00) was used for the detection of DA and serotonin (5-HT). The other (containing 50 mM Citric Acid.H_2_O, 25 mM NaH_2_PO_4,_ 1 mM EDTA, 1.4 mM octane sulfonic acid in 11.6% acetonitrile; pH adjusted to 4.15) was used to detect the DA metabolites (3,4-dihydroxyphenylacetic acid; DOPAC and homovanillic acid; HVA), and the 5-HT metabolite 5-hydroxyindoleacetic acid (5-HIAA). Detailed HPLC conditions and components are listed elsewhere^16^. For no-net flux calculations, [DA]_in_ was the amount of DA in the perfused DA standards. [DA]_out_ was the amount of DA in the dialysate output. [DA]_in_ – [DA]_out_ was plotted against [DA]_in_ to give a linear no-net flux regression for each animal. The x-intercept of this line gives a reliable estimate of baseline extracellular DA in the brain. KCl-induced DA release was analyzed both as raw pg/μl of DA, and as a % change from baseline (the average DA amount in the previous 2 baseline bins). The change in metabolite levels as a result of KCl infusion was assessed by comparing pre-KCl values (the average of baseline bins 3 and 4), with the post-KCl values (the average of the KCl infusion bin and post-KCl bin 1) using a paired *t*-test.

### Locomotor, rearing and stereotyped response to 5 mg/kg AMPH

The behavioural response to 5 mg/kg AMPH was assessed using the same open-field chambers and Ethovision software as for the repeated 0.6 mg/kg AMPH test. Stereotypies and rearing were manually quantified by a trained experimenter blind to group. The number of rears was counted for 5-min periods starting at 0, 30, and 60 min following AMPH administration. A rear was counted when the animal stood on its hind legs, and at least one forepaw touched a wall. To assess stereotypies, animals were scan-sampled for 10-min periods at 30–40 min and 60–70 min following AMPH administration. During every minute, the expression of a stereotyped behaviour—repetitive movements (such as repetitive sniffing and grooming, but not including circling), head weaving, or tight circling—was scored. Scores were summed to provide a total stereotypy score for each 10-min bin. A score of 10 indicates that an animal was engaged in a stereotyped behavior for the entire 10-min period, and this is the maximum score possible.

### Statistical analysis

All data were analysed using SPSS (IBM, ver. 26). Due to some missing samples, the repeat AMPH-induced locomotion and repeat PPI data was analysed using a linear mixed model with random intercepts, and a diagonal covariance matrix. Outliers (values outside 1.5 × the inter-quartile range) were excluded; for the microdialysis experiments, 1 EDiPS animal was excluded for DA, 1 control animal was excluded for DOPAC, HVA and 5-HIAA, and 1 control and 1 EDiPS animal were excluded for 5-HT. To assess differences in variability between baseline extracellular DA for EDiPS and control groups, we used the Brown-Forsythe test. Because of its skewed and ordinal nature, a non-parametric Mann–Whitney *t*-test was used to analyse the stereotypy scores. All other data were normally distributed. All other data were assessed with repeated measures ANOVA or *t*-tests as appropriate. Any significant findings were followed up post-hoc with *t*-tests, and a Bonferroni adjustment was performed to correct for multiple comparisons. Significance was assigned at *p* ≤ 0.05, and data are displayed with SEM.

## Results

### Longitudinal 0.6 mg/kg AMPH-induced hyperlocomotion

**Figure 2:**
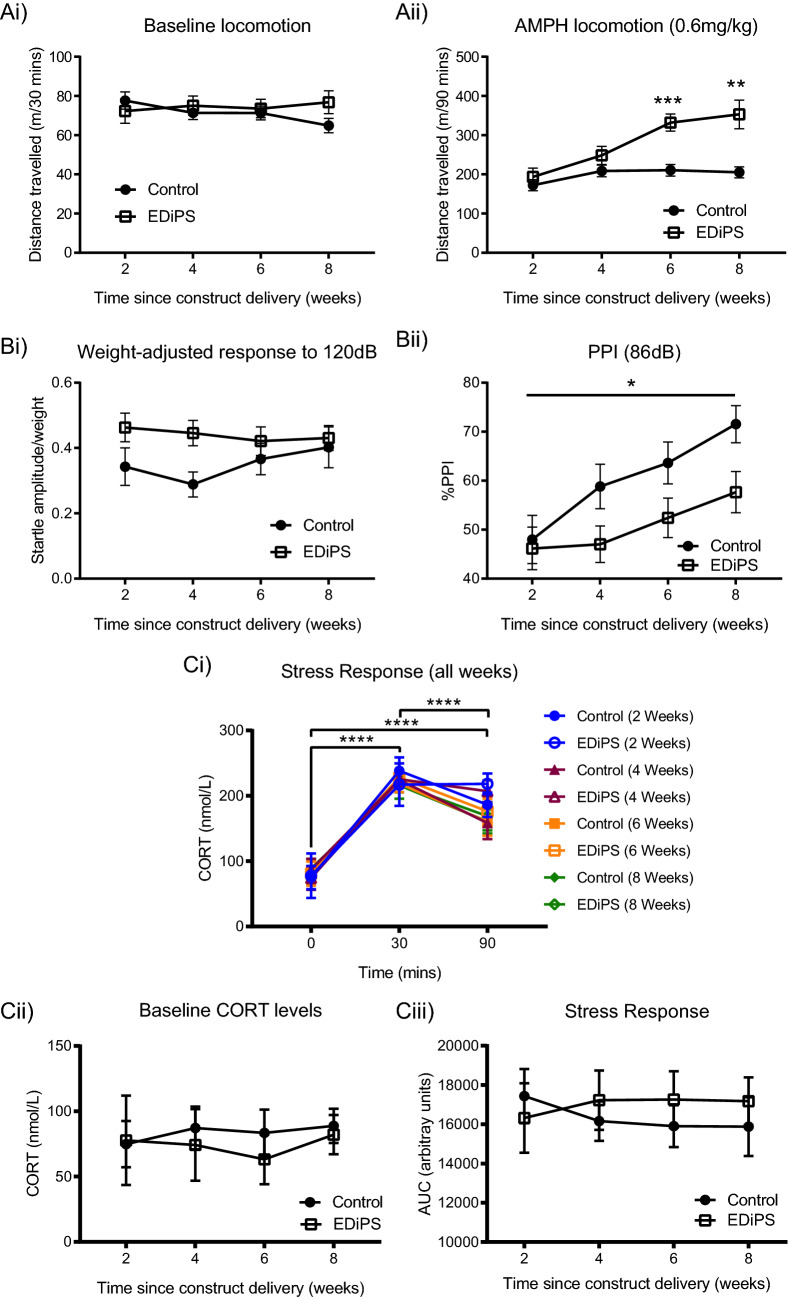
Longitudinal behavioural and stress response testing in EDiPS animals. (**Ai**) There was no difference between groups for baseline locomotion during the 30-min habituation period prior to AMPH administration. (**Aii**) There was an overall group effect of EDiPS on total distance travelled following 0.6 mg/kg AMPH, as well as significant week × EDiPS interactions at the 6- and 8-week timepoints. (**Bi**) EDiPS had no effect on ASR (adjusted for increasing animal weight over time). (**Bii**) EDiPS animals did show an overall deficit in %PPI. There was also a main effect of time such that %PPI increased across testing periods. (**Ci**) There was a significant increase in CORT levels 30 min following the onset of restraint stress, and a significant decrease by the end of the test, at 90 min, and this was true for all timepoints tested. (**Cii**) There was no difference between baseline CORT levels for EDiPS and control animals across the timepoints assessed. (**Ciii**) There was also no difference in the stress response (as indicated by area under the curve; AUC) for EDiPS and control animals across all four timepoints. *AMPH* Amphetamine, *ASR* acoustic startle response, *PPI* pre-pulse inhibition, *CORT* corticosterone. *p < 0.05, **p < 0.01, ***p < 0.001, ****p < 0.0001. ± SEM.

We first examined baseline locomotion prior to AMPH administration (Fig. 2Ai). We saw no effect of EDiPS (*F*_(1,28.9)_ = 0.22, *p* = 0.64), week (*F*_(3,15.7)_ = 0.53, *p* = 0.66), or a week × EDiPS interaction (*F*_(3,15.7)_ = 0.70, *p* = 0.56) for baseline locomotion. There was an effect of week on total distance travelled after 0.6 mg/kg AMPH (*F*_(3,18.65)_ = 111.8, *p* < 0.0001; Fig. 2Aii). We further found an overall effect of EDiPS (*F*_(1,25.6)_ = 13.3, *p* = 0.001), driven by a week × EDiPS interaction (*F*_(3,18.6)_ = 6.33, *p* = 0.004). Post-hoc analysis revealed that EDiPS animals moved more compared to controls at 6 weeks (*p* = 0.0004) and 8 weeks (*p* = 0.032) following construct delivery.

### Longitudinal PPI

We first established that there was no EDiPS (*F*_(1,14)_ = 2.45, *p* = 0.142) or week × EDiPS (*F*_(3,42)_ = 0.58, *p* = 0.62) effect on weight gain during adolescence (data not shown). The ASR to the 120 dB stimulus (adjusted for increasing body weight), did not vary with increasing age (*F*_(3,40.9)_ = 1.08, *p* = 0.36; Fig. 2Bi) and more importantly there was no effect of EDiPS (*F*_(1,39.8)_ = 1.82, *p* = 0.18) or a week × EDiPS interaction (*F*_(3,40.9)_ = 1.76, *p* = 0.17). We assessed PPI at a range of inter-stimulus intervals and intensities (full results are tabulated in Supplementary Table S1). In accordance with the literature, we saw pre-pulse facilitation at the short inter-stimulus interval (8 ms), and the lower stimulus intensity (74 dB)^27,28^. We also did not see an effect of week for these variables, whereas an effect of week was seen for all other inter-stimulus intervals and intensities (which did induce PPI), which aligns with the increase in PPI over adolescence as seen in the literature^29^. We saw a significant effect of EDiPS and a time × EDiPS interaction for the 64 and 128 ms inter-stimulus intervals (*p* = 0.049 and *p* = 0.045 respectively). The absence of significant findings for other inter-stimulus intervals may be due to the limited number of trials pooled (5) for each interval. However, when analysing the pre-pulse intensity which gave the greatest PPI effect in control animals (86 dB), we saw that there was an overall effect of EDiPS (*F*_(1,39.1)_ = 4.38, *p* = 0.043), but no week × EDiPS interaction (*F*_(3,48.6)_ = 1.27, *p* = 0.29; Fig. 2Bii). Similar differences between control and EDiPS groups were seen for the 78 dB pre-pulse intensity, which also results in robust levels of PPI.

### Longitudinal response to restraint stress in EDiPS animals

A repeat measures ANOVA was performed for CORT levels at the 0-, 30- and 90-min timepoints for all weeks and both groups (Fig. 2Ci). Levels of CORT significantly increased 30-min after the stress initiation, compared to t = 0 (baseline) (*p* < 0.0001). There was also a significant decrease in CORT levels compared to the 30-min timepoint by the 90-min collection time (*p* < 0.0001). This pattern is consistent with normal HPA function in response to acute restraint stress at these ages^30^. There was no effect of EDiPS (*F*_(1,10)_ = 0.15, *p* = 0.70) or week (*F*_(3,30)_ = 0.29, *p* = 0.82) on baseline CORT, nor a week × EDiPS interaction (*F*_(3,30)_ = 0.25, *p* = 0.85; Fig. 2Cii). This indicates no change in basal stress levels as a result of repeat testing. To assess the response to restraint stress, we analysed the area under the curve (AUC) for each animal at each timepoint (Fig. 2Ciii). Again, there was no effect of week (*F*_(3,33)_ = 0.04, *p* = 0.98), EDiPS (*F*_(1,11)_ = 0.17, *p* = 0.68) or a week × EDiPS interaction (*F*_(3,33)_ = 0.67, *p* = 0.57).

### Baseline extracellular DA and KCl-induced DA release in adult EDiPS animals

**Figure 3:**
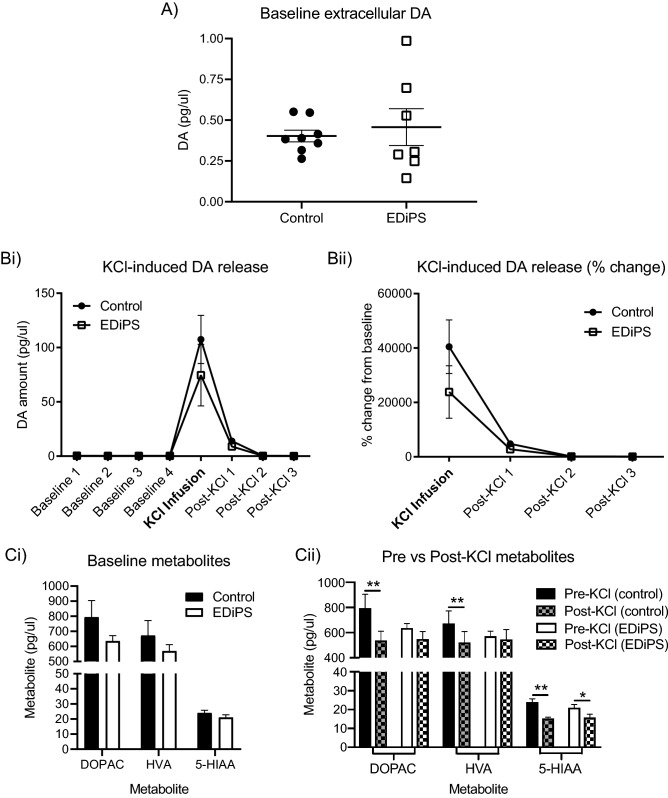
Baseline and KCl-evoked DA and metabolites. (**A**) There was no significant difference between EDiPS and control groups for baseline extracellular DA. (**Bi**) KCl-evoked DA release was normal in EDiPS animals when analysed either as total amount or (**Bii**) as a % change from baseline. (**Ci**) A regular 2-way ANOVA of the baseline levels of each metabolite (the average of the last 2 baseline bins—baseline 3 and baseline 4) indicated no difference for any metabolite between EDiPS and control animals. (**Cii**) Metabolites were also compared pre- and post-KCl infusion. Control animals showed the expected decline in metabolite levels following KCl infusion, whereas this was only evident in EDiPS animals for the serotonin metabolite 5-HIAA. *ASR* acoustic startle response, *DOPAC* 3,4-dihydroxyphenylacetic acid, *HVA* homovanillic acid, *5-HIAA* 5-hydroxyindoleacetic acid. *p < 0.05, **p < 0.01. ± SEM.

Baseline extracellular DA levels were significantly more variable in the EDiPS group compared to the control group (*F*_(6,7)_ = 8.635, *p* = 0.011). We found no significant difference between EDiPS and control animals for baseline extracellular DA (*t*_(7.2)_ = 0.46, *p* = 0.66, with Welch’s correction; Fig. 3A). There was also no difference between EDiPS and control animals for the slope of the no-net flux regression (*F*_(1,71)_ = 1.59, *p* = 0.21). Infusion of the high potassium (KCl) solution resulted in similarly increased levels of evoked DA release for both EDiPS and control animals (*t*_(14)_ = 0.64, *p* = 0.52; Fig. 3Bi). There was also the case when this data was analysed as % change from baseline (the average of baselines 3 and 4) (*t*_(14)_ = 1.36, *p* = 0.19; Fig. 3Bii). There was no difference in baseline extracellular levels of 5-HT (*t*_(11)_ = 0.98, *p* = 0.34), or the amount of 5-HT released following KCl (*t*_(11)_ = 0.4, *p* = 0.69) between EDiPS and control animals (data not shown). EDiPS had no effect on baseline extracellular levels of DOPAC, HVA, or 5-HIAA (*F*_(1,27)_ = 3.2, *p* = 0.08; Fig. 3Ci). Levels of these metabolites were also not significantly different between groups following KCl infusion, however control animals showed the normal decrease in all metabolites expected after the high potassium solution (DOPAC; *p* = 0.003, HVA; *p* = 0.043, 5-HIAA; *p* = 0.0037), whereas in EDiPS animals this was only seen for the 5-HT metabolite, 5-HIAA (*p* = 0.018; Fig. 3Cii).

### Behaviour following 5 mg/kg AMPH

**Figure 4:**
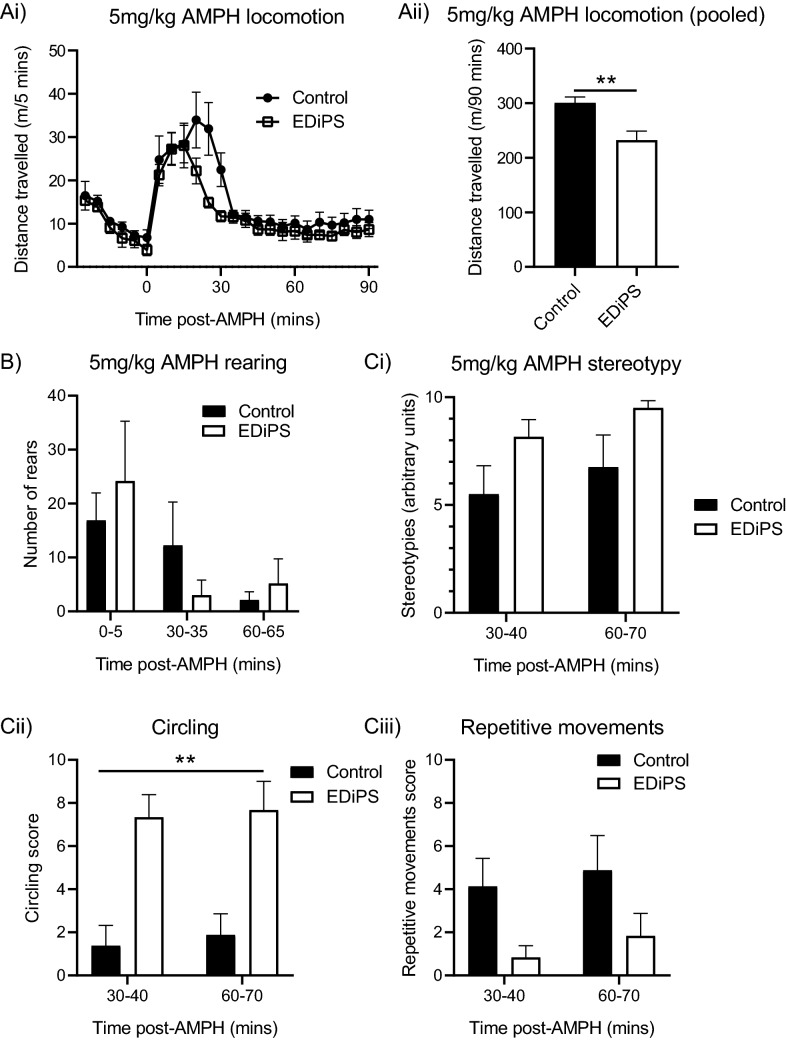
Behavioural responses to 5 mg/kg AMPH. (**Ai**,**Aii**) EDiPS animals showed significantly reduced locomotion in response to 5 mg/kg AMPH compared to control animals. (**B**) The number of rearing bouts post-AMPH was not different between EDiPS and control animals. (**Ci**) EDiPS animals did show a visible trend of increased stereotyped behaviour following 5 mg/kg AMPH, however this was not statistically significant. (**Cii**) Although overall stereotypy scores were unaltered between EDiPS and control animals, EDiPS animals displayed significantly more circling behaviour compared to control animals. (**Ciii**) The number of repetitive movements performed was statistically unchanged in EDiPS compared to control animals. *AMPH* Amphetamine. **p < 0.05. ± SEM.

EDiPS animals travelled less distance following 5 mg/kg AMPH compared to control animals (*t*_(12)_ = 3.46, *p* = 0.0061; Fig. 4Ai,ii). A repeat measures ANOVA revealed that there was no difference in the number of rears performed in the two 5-min bins assessed following AMPH administration (*F*_(1,12)_ = 0.27, *p* = 0.61; Fig. 4B). There was no difference in overall stereotypy scores between EDiPS and control animals during either the 30–40 min period (*U* = 13.5, *W* = 49.5, *z* = − 1.36, *p* = 0.18) or the 60–70 min period (*U* = 16.5, *W* = 52.5, *z* = − 1.07, *p* = 0.34; Fig. 4Ci). Although overall stereotypy scores were not different, when these scores were separated into specific behaviours, EDiPS animals displayed significantly more circling behaviour (*F*_(1,12)_ = 17.4, *p* = 0.001; Fig. 4Cii) compared to controls. The number of repetitive movements performed was unaltered in EDiPS animals compared to controls (*F*_(1,12)_ = 4.26, *p* = 0.06; Fig. 4Ciii).

## Discussion

**Figure 5:**
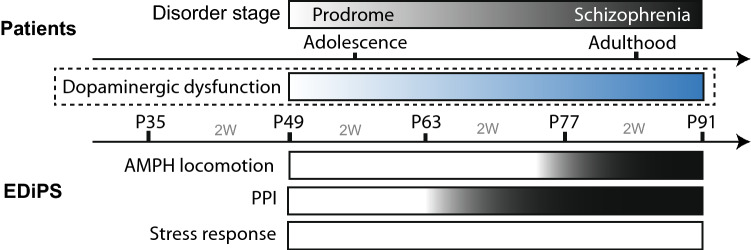
Summary of findings. In EDiPS animals, the increase in AMPH-induced hyperlocomotion is evident from 6 weeks following administration of the EDiPS construct at P35. In contrast, the PPI deficit is apparent from 4 weeks following administration of the construct. No difference in the response to stress is seen over this time. This progression of behavioural phenotypes likely reflects an increasing dysfunction of dopaminergic activity in the dorsal striatum, induced by the EDiPS construct. This parallels the increase in dopaminergic dysfunction which appears to contribute to the transition in patients from the prodromal stage of schizophrenia to expression of the full disorder.

The findings from this study suggest that the behavioural phenotypes relevant to the positive symptoms of schizophrenia in EDiPS emerge progressively during adolescence (Fig. 5). Adult EDiPS animals have greater variance in baseline extracellular DA levels, however the absence of a mean difference compared with controls suggests that tonic DA may be normal. Vesicular DA content also appears to be normal.

We have previously established that increased AMPH-mediated locomotion and a deficit in PPI are evident in adult EDiPS animals^16^. Here we replicate those findings and show that the onset of these phenotypes is progressive. The effects of AMPH on locomotion become significant only from 6 weeks following administration of the EDiPS construct. While these findings may indicate that EDiPS animals are becoming sensitised to repeated AMPH administration, comparison to previous locomotor data at the 8-week timepoint^16^ indicates no difference in response to AMPH whether animals had been exposed to prior doses of AMPH or not (see Supplementary Fig. S2). Additionally, the low dose of AMPH used here, and the long intervals between treatments make such sensitisation highly unlikely. It is also worth noting that AMPH-induced hyperlocomotion has been typically associated with DA release in the NAc, rather than the DS. This issue was discussed in our original paper on the EDiPS model^16^. Firstly, there is some evidence that DA in the DS can modulate locomotor activity^31^. Secondly, even nigro-striatal-specific delivery of the EDiPS construct may affect accumbal DA, due either to effects of the construct within the small number of neurons in the pars compacta which project to the nucleus accumbens^19^, or to feedback mechanisms through the basal ganglia from the DS to the NAc^32^. This aspect of the EDiPS model requires continued consideration.

EDiPS animals showed an overall deficit in PPI. Although no statistically significant timepoint × EDiPS interaction was evident, this overall deficit appears to be driven by the difference in EDiPS and control animals from 4 weeks onwards. There was also a significant effect of age on %PPI for both groups indicating that, as expected, the magnitude of PPI increases across adolescence into adulthood^29^. The frequency and intensity of psychotic symptoms generally increases as patients transition from the prodrome to schizophrenia^33^. When assessed longitudinally, PPI deficits also become worse in ARMS patients who transition to clinical schizophrenia^34^. Therefore, the progression of the behavioural phenotypes in EDiPS animals suggest that it could be an ideal model to examine the presynaptic dopaminergic mechanisms governing transition from the prodrome to schizophrenia.

These findings suggest that the effects of the EDiPS construct are not fully established between 5 and 9 weeks of age (equivalent to the peri-pubertal period in Sprague–Dawley male rats^35,36^) but become apparent by early adulthood (approximately P70). This pattern of phenotype progression is roughly equivalent to the onset of schizophrenia^5^. Immunohistological analysis indicates that, at the 2-week time-point, construct-generated proteins are localised in the midbrain, but not the striatum. At the 8-week timepoint, they are apparent in both regions, indicating a delay in the transport of construct-generated mRNA/protein from the midbrain to the dorsal striatum. Therefore, the absence of behavioural deficits in EDiPS animals at this time-point is likely due to the lack of construct expression in the target region—the dorsal striatum. The delayed onset of behavioural deficits therefore likely reflects a combination of progressive construct expression in the dorsal striatum, and its interactions with the maturing dopaminergic system.

The deficit in PPI and the increase in AMPH-induced hyperlocomotion are evident 4- and 6-weeks after construct delivery respectively. This temporal difference may be due to the complex interaction between the effects of the EDiPS construct and normal dopaminergic maturation. While axonal innervation from the nigra to the DS is complete by approximately P14^37^, DA levels in the brain increase until adulthood^38^. Levels of the D1- and D2-type DA receptors increase until P28–P40 and then decline^39–41^, suggesting a toning of the DA response in the striatum over this time. An artificial increase in DA synthesis capacity in adolescence induced by the EDiPS construct may lead to abnormally precocious modulation of the DA system. Consideration of the similarly maturing meso-cortical and meso-limbic dopaminergic circuits^42^, as well as the concurrent maturation of other neurotransmitter systems^43^ would be required to fully understand this phenotypic pattern.

Contrary to our expectations, EDiPS animals did not differ in their physiological response to repeated physical restraint stress. In patients, a psychosocial stressor—the Montreal Imaging Stress Task (MIST)—is widely used to induce a stress response^44^. A PET study revealed that both patients with chronic schizophrenia and ARMS patients show increased DA release in the DS when subjected to the MIST, compared to healthy controls^45^. Whether EDiPS animals who are primed to synthesise more DA in the DS would display increased DA release to an equivalent psychological stressor is now a subject for future investigation.

We predicted that EDiPS animals would show increased baseline extracellular DA, consistent with the increased DA synthesis capacity in this model^16^. However, baseline extracellular DA levels and KCl-evoked DA release were unaltered between EDiPS and control animals. Therefore, although the capacity to synthesize DA is increased in EDiPS animals, this does not necessarily result in increased baseline extracellular DA. A number of mechanisms exist to regulate pre-synaptic DA content. Phosphorylation is a key determinant of TH enzymatic activity^46^, and binding of DA to D2 autoreceptors on the presynaptic terminal can act to decrease DA synthesis by reducing TH phosphorylation^47^. Autoreceptor binding can also decrease vesicular release of DA^48^. Finally, DA itself can inhibit activity of the TH enzyme by binding to the active site and inducing a conformational change in the structure of the enzyme^49,50^. It is also possible that basal ganglia feedback loops have been recruited to normalise the increased DA synthesis in the DS^32,51^. The complexity of the basal ganglia circuit limits any speculation on this mechanism, since we currently only have data from the DS.

A high potassium solution normally leads to decreased extracellular levels of DOPAC and HVA^52^. This reduction in DA metabolites was less pronounced in EDiPS animals compared to control animals. A decline in DOPAC is interpreted as a reduction in DA available as a substrate within the pre-synaptic cytosol, due to increased DA release into the synapse^52^. Importantly, DOPAC is believed to be predominantly generated from newly synthesized DA^53^. Extracellular HVA levels reflect the competition between decreased intracellular synthesis (as for DOPAC), and increased extracellular synthesis as a result of increased available synaptic DA^54^. Therefore, in EDiPS animals, the normal decline of these metabolites after chemical depolarization is likely prevented by the increased capacity to generate DA. This is further evidence that, while baseline extracellular DA may be unaltered in EDiPS compared to control animals, aspects of pre-synaptic DA activity are abnormal in this model.

EDiPS does not appear to recapitulate the increased baseline synaptic DA which has been found in patients with schizophrenia^55–57^. However, it does replicate the recent meta-analysis finding of greater variance in synaptic DA found in patients compared to controls^10^. In EDiPS animals, this variability may simply reflect experimental differences in the placement of the construct. However, the spread of EDiPS values extends both below as well as above the control values, suggesting some other clinically relevant source of biological variability. Investigating this variability in EDiPS animals may be critical to understanding the clinical and biological heterogeneity evident in schizophrenia.

Despite overall stereotypy scores being unaltered between EDiPS and control animals, EDiPS animals showed a preference for tight circling in comparison to repetitive behaviours such as repeated sniffing and grooming. Circling behaviour can indicate a hemispheric imbalance of DA activity^58,59^. The EDiPS construct is administered bilaterally, and the amount of TH and GCH1 expressed in each hemisphere is likely to be slightly unequal, reflecting marginal differences in the volume and placement of the construct. This circling behaviour is therefore a highly probable response of EDiPS animals to high-dose AMPH.

## Limitations

A key limitation in this study is the absence of neurobiological assessment of dopaminergic dysfunction alongside the behavioural phenotypes. Ideally this would be performed longitudinally, however long-term microdialysis is not feasible. Fast scan cyclic voltammetry (FSCV) is another technique used to measure DA in vivo. A recent paper showed that, using a smaller, flexible FSCV probe, it is possible to acquire DA measurements for more than 1 year after its implantation in the rodent brain^60^. This technique is still in its infancy and requires replication. However, understanding the progression of dopaminergic dysfunction in EDiPS animals, especially alongside normal adolescent maturation, is crucial, and will be a focus of future studies. A further limitation is the use of only male animals in these experiments. While this was chosen to minimise the number of animals used, we plan to include female animals in future experiments.

## Conclusion

These results indicate the robustness of the behavioural phenotypes seen in this model; we replicate a PPI deficit and increase in AMPH-induced hyperlocomotion in EDiPS animals. We now demonstrate that these behavioural abnormalities show a progressive onset, which may have relevance to ongoing alterations in DA circuitry during the schizophrenia prodrome. Now, we can unpick the mechanism behind these phenotypes, particularly in the context of normal brain development over adolescence. EDiPS is therefore a valuable model which might improve our understanding of disease course, and could be critical for trialling potential therapies for intervention.

## Supplementary Information

Supplementary Information.
