# Supplementary material for: Positive symptom phenotypes appear progressively in “EDiPS”, a new animal model of the schizophrenia prodrome
Source: Sci Rep. 2021 Feb 22;11:4294. doi: 10.1038/s41598-021-83681-4 (PMC7900200; doi:10.1038/s41598-021-83681-4)
Supplement: Supplementary file 1 — Supplementary Information. [file 41598_2021_83681_MOESM1_ESM.pdf]

Positive symptom phenotypes appear progressively in “EDiPS”, a new animal model of the schizophrenia prodrome

Dr Alice Petty[1,2]., Dr Xiaoying Cui[1]., Dr Asad Ali[1]., Mr Zilong Du[1]., Mr Sunil Srivastav[1]., Dr James Kesby[1], Prof Deniz Kirik[3]., Prof Oliver Howes[2,4,5]., Prof Darryl Eyles[1,6]*.

Supplementary Material

Construct expression in five separate animals 2- weeks post-delivery compared with an adult animal at 8-weeks post-construct delivery:

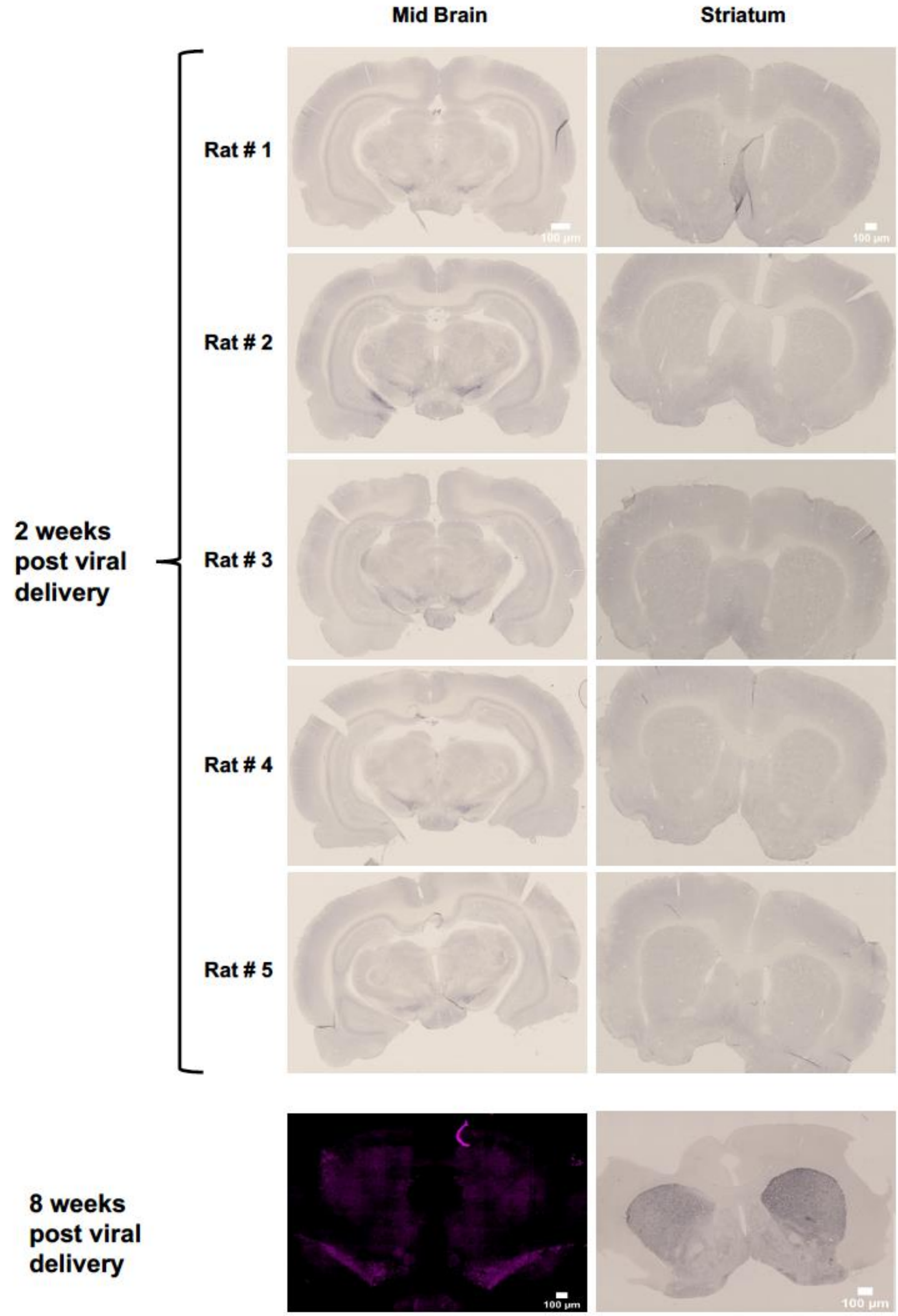

Figure S1. Representative images from immunohistochemical visualisation of human (hu)GCH1 in a midbrain and striatum. Sections were taken from five separate animals two weeks post EDiPS construct delivery. huGCH1 can be used to localise construct placement, since the endogenous rat GCH1 is not recognised by this antibody (Cederfjall, et al., 2013). *Immunohistochemistry:* In the five animals who were 2 weeks post-construct delivery and the adult striatum which is eight weeks post-construct delivery, brains were sectioned in a 1-in-6 series at 40um using a cryostat. Immunohistochemistry was used to confirm construct expression both at the injection site (SNpc) and the DS target. Immunohistochemistry was performed using a primary antibody raised against huGCH1 (HPA028612, polyclonal rabbit IgG, 1:250, Sigma). huGCH1 was visualised using a Goat anti-rabbit IgG antibody, biotinylated (Vector Laboratories, Burlingame, CA, USA, Cat#BA1000-1.5), 1:1000 and a di-amino benzadine (DAB) nickel technique according to the manufacture's protocol. We also show huGCH1 in midbrain from an adult animal (8 weeks post-construct) administration using the same primary antibody and a fluorescent secondary Alexa Fluor 488 goat anti-rabbit, (Life Technologies, 1:500). There is clear expression of huGCH1 in the substantia nigra of all five EDiPS animals two weeks following delivery of the construct into the brain, however, no such expression is evident in the striatum at this age. In a comparative mid brain and striatum from adult animals eight weeks following construct delivery, huGCH1 expression is clearly evident in both the midbrain and striatum.

Absence of sensitisation to amphetamine in EDiPS animals:

In our previous publication (Petty et al., 2019), we administered amphetamine (AMPH) at a single timepoint, 8 weeks following construct delivery. In the current study, we administered AMPH at 4 timepoints, 2, 4, 6, and 8 weeks following construct delivery. The dose administered (0.6mg/kg) was identical between these studies, as were all aspects of the protocol and apparatus used (30 minutes habituation prior to AMPH administration, black matt 60x60x60 chambers). In both studies, analysis of locomotor activity was performed using Ethovision software. We found no difference for distance travelled at the 8-week timepoint between EDiPS animals which had been administered AMPH at that single timepoint, or repeatedly prior to that timepoint ($t_{(16)}$=0.65, p=0.52; Fig. S2). This strongly suggests the absence of sensitisation of EDiPS animals to repeated administration of AMPH. If sensitisation had occurred, we would expect an increase in locomotor activity at the 8-week timepoint for those animals which had received AMPH previously, compared to those which received a single dose.

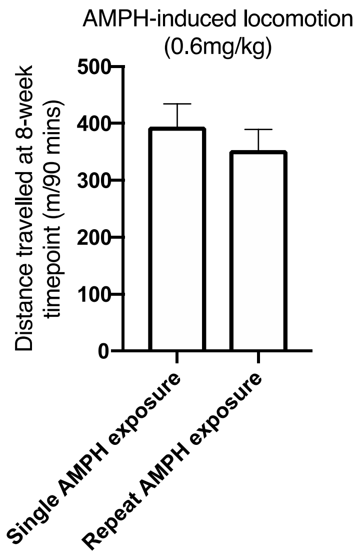

Figure S2. Locomotor response of EDiPS animals to either a single, or repeated administration of AMPH. There was no significant difference between the locomotor response of EDiPS animals to AMPH at 8 weeks following construct delivery (p=0.52), regardless of whether they had been exposed to AMPH at prior timepoints or not. Note that the single AMPH exposure data comes from previously published work (Petty et al., 2019). This data strongly suggests that EDiPS animals did not sensitise to repeated AMPH administration at 2, 4, 6 and 8-weeks following construct delivery. AMPH; amphetamine. ± SEM.

Table S1.

Week		Inter-stimulus interval (ms)						Pre-pulse intensity (dB)		
		**8**	**16**	**32**	**64**	**128**	**256**	**74**	**78**	**86**
**2**	Control (n=14)	-8.8 (19.5)	9.8 (9.2)	29.7 (6.9)	42.5 (5.5)	15.4 (8.3)	17.4 (8.7)	-27.2 (10.4)	29.1 (5.8)	47.9 (5.2)
	EDiPS (n=16)	0.02 (8.1)	6.5 (6.2)	32.9 (6.2)	33.6 (7.1)	30.5 (5.1)	31.7 5.6)	-4.9 (9.7)	26.5 (4.2)	46.1 (4.7)
**4**	Control (n=18)	-3.02 (10.2)	12.6 (5.5)	45.6 (6.2)	52.0 (4.4)	30.6 (6.3)	28.3 (9.2)	-10.8 (9.2)	36.1 (6.1)	57.7 (4.9)
	EDiPS (n=20)	-4.8 (7.5)	10.8 (6.2)	44.6 (4.5)	43.3 (4.9)	28.1 (4.8)	28.2 (4.3)	0.9 (5.4)	28.2 (4.4)	46.0 (3.8)
**6**	Control (n=20)	6.4 (9.1)	14.1 (9.1)	53.2 (5.0)	58.1 (3.1)	39.9 (6.7)	40.6 (5.4)	-2.5 (9.9)	45.2 (4.6)	63.1 3.8)
	EDiPS (n=21)	-5.1 (8.0)	9.9 (5.5)	46.4 (5.6)	47.2 (4.3)	27.1 (5.8)	29.0 (4.3)	-5.5 (5.6)	29.8 (5.5)	53.0 (3.9)
**8**	Control (n=18)	5.7 (11.4)	34.7 (6.6)	64.6 (4.8)	64.1 (4.3)	46.4 (7.2)	47.6 (6.1)	7.6 (11.0)	52.3 (5.4)	71.5 3.7)
	EDiPS (n=20)	10.1 (5.8)	15.7 (6.1)	49.6 (5.6)	50.5 (4.7)	32.9 (5.9)	33.3 (6.1)	1.5 (6.1)	36.9 (5.4)	57.6 (4.2)
**Effect of time (*p*)**		0.57	0.022*	0.0001****	0.0001****	0.026*	0.021*	0.47	0.001***	0.0001****
**Effect of group (*p*)**		0.94	0.22	0.337	0.049*	0.52	0.64	0.66	0.038*	0.043*
**Time*group interaction (*p*)**		0.69	0.37	0.268	0.879	0.045*	0.071	0.59	0.344	0.29

Mean pre-pulse inhibition (%) at the range of inter-stimulus intervals and pre-pulse intensities tested. (SEM). Analysis was performed using a linear mixed effects model, with details in the main text.

Cederfjall, E., Nilsson, N., Sahin, G., Chu, Y., Nikitidou, E., Bjorklund, T., Kordower, J. H & Kirik, D. (2013). Continuous DOPA synthesis from a single AAV: dosing and efficacy in models of Parkinson's disease. Sci Rep, 3, 2157.

Petty, A., Cui, X., Tesiram, Y., Kirik, D., Howes, O & Eyles, D. (2019). Enhanced Dopamine in Prodromal Schizophrenia (EDiPS): a new animal model of relevance to schizophrenia. *NPJ Schizophr, 5*(1), 6. doi:10.1038/s41537-019-0074-z
